# Case report: Torpedo maculopathy in a pediatric patient

**DOI:** 10.22336/rjo.2021.77

**Published:** 2021

**Authors:** Alina Dumitru, Daniela Goicea, Irina Ștefan

**Affiliations:** * Focus Optic Clinic, Bucharest, Romania

**Keywords:** torpedo maculopathy, pediatric patient, optical coherence tomography, retinal pigment epithelium

## Abstract

**Objective:** Torpedo maculopathy (TM) is an atypical congenital retinal lesion and, as the name suggests, with an oval shape like a torpedo, usually situated in the temporal sector of the macula. The objective of this paper was to show an accidental finding of torpedo maculopathy in a pediatric patient.

**Case presentation:** A 7-year-old dizygotic twins Caucasian girl, born at 38 weeks, came to our clinic for a routine ophthalmological examination. She had no ocular history, other symptoms, pain, or decreased visual acuity in her left eye. The best-corrected visual acuity (BCVA) was 6/ 6 for both eyes. The posterior pole at OD (oculus dexter) was normal, without any pathological changes. OS (oculus sinister) fundus had an oval well defined hypopigmented lesion in the temporal sector of the macula, with the tip oriented towards the central fovea and the tail extending to the temporal retina, elongated in the horizontal axis, with normal underlying choroidal details, approximately 2-disc diameters (DD) in width and 1 DD in height, and with 2 smalls areas of hyperpigmentation at the level of temporal margin. The optical coherence tomography (OCT) realized on OS exposed an absent retinal pigment epithelium (RPE) combined with hyper-reflectivity of the adjacent choroid, a decreased thickness of ellipsoid zone (EZ), interdigitation zone (IZ), and the outer nuclear layer (ONL), with no focal choroidal cavitation. The inner retina was normal. OCT performed on OD was normal, without any pathological change.

**Conclusion:** Generally, torpedo maculopathy is an asymptomatic benign congenital retinal lesion that is most often diagnosed at a routine eye examination, but with an increased risk of choroidal neovascular membrane appearance, which is why the patients must have a long-term follow-up.

**Abbreviations:** TM = torpedo maculopathy, BCVA = best-corrected visual acuity, OD = oculus dexter, OS = oculus sinister, DD = disc diameters, OCT = optical coherence tomography, RPE = retinal pigment epithelium, EZ = ellipsoid zone, IZ = interdigitation zone, ONL = outer nuclear layer

## Introduction

Like the name suggests, torpedo maculopathy (TM) is a torpedo-shaped hypo-pigmented lesion in the temporal sector of the macula, which was initially reported in 1992 by Rosemann and Gass. Although the etiology of TM remains unidentified, there are some base theories that provide some possible origins of this pathogenesis such as “anomalies in the development of neuro-ectoderm” [**1**], “intra-uterine choroiditis” [**2**], “a developmental deficiency in the nerve fiber layer along the horizontal raphe” [**3**], or “persistent developmental defect in the RPE of the fetal temporal bulge” [**4**].

Currently, we do not have any clear statistical data on the incidence and prevalence of TM, but Shirley et al. estimated an incidence of 2/ 100.000 in Northern Ireland [**5**], which is probably underestimated, because typically, the patients are asymptomatic and these lesions are discovered during a routine ophthalmological examination.

There are reports with some association between TM and systemic disorders like blepharophimosis, kidney disease [**6**], tuberous sclerosis [**7**], or situs inversus [**3**]. 

## Case report

We report a case of a 7-year-old dizygotic twins Caucasian girl, born at 38 weeks, who came to our clinic for a routine eye examination. She had no ocular history, other symptoms, pain, or vision loss in her left eye. The patient and the parents denied any trauma history. The best-corrected visual acuity (BCVA) was 6/ 6 for both eyes. Extraocular motilities were full and smooth and, at cover-test, the patient presented orthophoria at distance and near. At the anterior segment, at OS (oculus sinister), the lens had small opacities in the nucleus of the lens and, at the OD, the lens was clear, the cornea was clear at OU (oculus uterque), pupils were round, equal, and responsive to light with no afferent pupillary defect. The intraocular pressure (IOP) was 14 mmHg at OU. The posterior pole at OD was normal, without any pathological changes of retina, optic nerve, or blood vessels. OS fundus had an oval well delineated hypopigmented lesion in the temporal sector of the macula, with the tip oriented towards the central fovea and the tail elongated to the temporal retina, extended in the horizontal axis, with normal underlying choroidal details, approximately 2-disc diameters (DD) in width and 1 DD in height, and with 2 smalls areas of hyperpigmentation at the level of temporal margin. Amsler grid test was unsuccessful and did not reveal any scotoma or metamorphopsia due to a poor understanding of age. Her brother was also consulted in our clinic without any pathological findings.

**Fig. 1:**
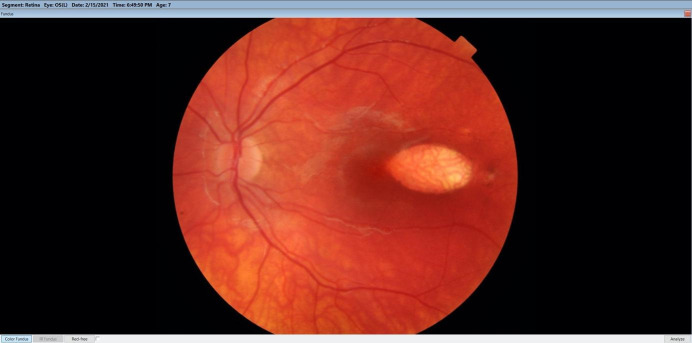
Color fundus photography of left eye showed an oval hypopigmented lesion on the temporal sector of the macula with 2 focal peripheral areas of hyperpigmentation at the level of temporal margin

**Fig. 2:**
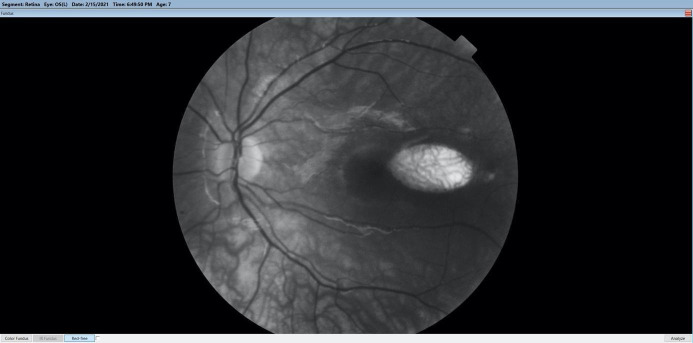
Red-free image of the depigmented retinal lesion

**Fig. 3:**
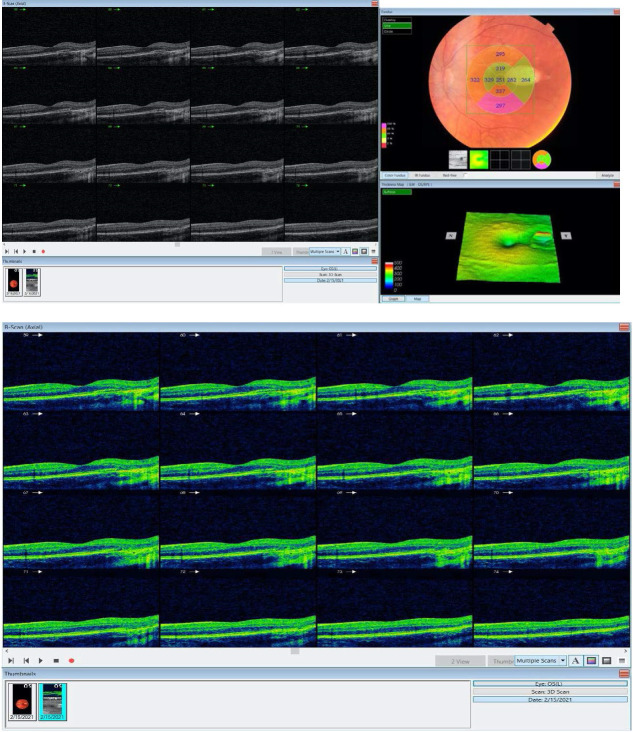
OCT (Topcon 3D 2000) performed on left eye showed an absent RPE combined with hyper-reflectivity of adjacent choroid, a decreased thickness EZ and IZ and ONL, without focal choroidal cavitation. The inner retina was normal. OCT performed on OD was normal, without any pathological change

## Discussion

In 1971, Donald Schlernitzauer presented in his report 2 cases of retinal pigment abnormality in which the cells of RPE were smaller in size at the site of the lesion due to a lack of the pigment granules and the edges of the lesion appeared to have the lancet shape. He presumed that the lesions are congenital rather than degenerative and the term used for this type of lesions was “peripheral retinal albinotic spot” [**8**].

In 1992, Gass and Roseman described “an oval placoid lesion with a wedge-shaped tail in the macula of the left eye of a 12-year-old boy” and they also presumed that was a congenital RPE lesion and they called it solitary “hypopigmented nevus” of RPE [**9**].

The term “torpedo maculopathy” was first introduced by Daily in 1993 and it described the lesion as congenital, which pointed toward the macula and was always localized in the temporal fovea [**10**].

The etiology of torpedo maculopathy remains unidentified, but there are some theories that have arisen over time. For example, “neuroectodermal theory”, supported by Klein [**1**], is based on some histological evidence that shows a faulty differentiation and an imbalance of growth of the choriocapillaris and pigment epithelium. Another hypothesis, according to Mann [**2**], is that of intra-uterine choroiditis, in which the appearance of the lesion depends on the nature and the timing of the infection during fetal development.

Also, another developmental theory supported by Pian et al. is that of “incomplete closure in the nerve fiber layer along the horizontal raphe” [**3**].

Based on Streeten’s study from 1969, Shields et al. [**4**] claimed that the reason for the TM “is a persistent defect in the development of RPE in the fetal temporal bulge”.

Currently, Wong et al. [**11**] categorized torpedo maculopathy, based on OCT appearance, into two types of lesions according to the presence of outer retinal and/ or choroidal excavation. Thereby, in type 1 of TM the inner retina appears normal and outer retina shows attenuation of IZ and EZ, decreased thickness of ONL, without retinal or inner choroidal excavation. Type 2 was established as having a normal inner retina, disappearance of outer retinal structures and thinning of ONL, to which outer retinal cavitation is included, which may or may not be accompanied by inner choroidal excavation. Torpedo lesions that contain choroidal excavation were lately proposed as “type 3” by Tripathy et al. [**12**], but with 2 conditions: to rule out other lesions that include excavated macular lesion and to have typical appearance of TM.

In their series of clinical cases, Venkatesh R et al. [**13**] described two varieties of torpedo lesions: those that involve the macula and those that do not. For those that do not affect the macula, it is important to make a differential diagnosis with other congenital pathologies of the RPE, like congenital hypertrophy of RPE (CHRPE), RPE lesions noticed in disease like Gardner syndrome and familial adenomatous polyposis, toxoplasma scar and traumatic injury.

Regarding our case, we found a close resemblance in terms of clinical and imaging features of TM. According to literature, the incidence of choroidal neovascularization (CNV) is exceptional and an optical coherence tomography angiography (OCTA) could help discover a potential appearance of choroidal neovascular membrane. Besides OCTA, microperimetry could detect microscotoma, which would not be detected by standard perimetry methods. It is considered that these microscotoma that could occur, result from reduced metabolites and oxygen supply for the inner retina and secondary loss of choriocapillaris and degeneration of photoreceptor so that the retinal sensitivity decreases [**14**]. Venkatesh et al. [**12**] performed adaptive optics imaging for two patients with TM, using Rtx1 flood illumination-based fundus camera, and they observed a reduced cone thickness and an increased space between the cells in the region of the lesion. But, for the cases of TM detected in the pediatric population, these paraclinical investigations could be difficult to perform either due to poor collaboration and understanding of age, or due to inaccessibility to these investigations like in our case.

## Conclusion

We described a case of TM in a 7-year-old dizygotic twins girl, born at term, without any associated pathologies. Generally, TM is an asymptomatic benign congenital retinal lesion, which is most often diagnosed at a routine eye examination, but with an increased risk of choroidal neovascular membrane appearance. This is the main reason why patients must have a long-term follow-up and be aware to report visual symptoms.

**Conflict of Interest statement**

The authors declare no conflict of interest.

**Informed Consent and Human and Animal Rights statement**

Informed consent has been obtained from all individuals included in this study.

**Authorization for the use of human subjects**

Ethical approval: The research related to human use complies with all the relevant national regulations, institutional policies, is in accordance with the tenets of the Helsinki Declaration, and has been approved by the review board of Focus Optic Clinic, Bucharest, Romania.

**Acknowledgments**

None.

**Sources of Funding**

None.

**Disclosures**

None.
